# The Effect of External Cold and Vibration on Infiltration-Induced Pain in Children: A Randomized Clinical Trial

**DOI:** 10.1155/2022/7292595

**Published:** 2022-09-05

**Authors:** Reihaneh Faghihian, Mona Esmaeili, Hossein Asadi, Mohammad Hossein Nikbakht, Fateme Shadmanfar, Mehdi Jafarzadeh

**Affiliations:** ^1^Dental Research Center, Department of Pediatric Dentistry, Dental Research Institute, Isfahan University of Medical Sciences, Isfahan, Iran; ^2^Dental Research Center, Department of Pediatric Dentistry, School of Dentistry, Isfahan University of Medical Sciences, Isfahan, Iran; ^3^Student Research Committee, School of Dentistry, Isfahan University of Medical Sciences, Isfahan, Iran

## Abstract

**Introduction:**

Children's fear of and anxiety about dental treatments are important problems in maintaining health. The anesthetic injection is the main cause of dental fear. One of the methods to reduce the infiltration-induced pain is to use external cold or vibration using the gate control system. Various devices have been used to apply cold and vibration, including the BUZZY device (BUZZY Company, Arizona). Studies have shown contradictory results for the effectiveness of cold and vibration. This study aimed to investigate the effect of cold and vibration versus cold alone on maxillary infiltration-induced pain and stress.

**Methods:**

Thirty children aged 6–12 years who required profound restoration of deciduous or permanent first molars were recruited in this randomized double-blind clinical trial. The anesthetic gel and BUZZY device were used in half of the children's jaws, and the anesthetic gel and the cold alone were used in the other half of the jaws. To measure stress from the heart rate, the Wong–Baker scale was used as the subjective scale, and the face, legs, activity, cry, consolability (FLACC) scale was used as the objective scale.

**Results:**

The FLACC score was significantly lower in the BUZZY group than in the cold-alone group, but the Wong–Baker scale and heart rate did not show a significant difference between the two groups.

**Conclusions:**

The BUZZY device can be effective in reducing infiltration-induced dental pain.

## 1. Introduction

Children's fear and anxiety about dental treatments are from significant health challenges. Research on children has shown that the prevalence of fear and anxiety varies from 7.5 to 21%, depending on the dental procedure [1]. Dental fear exists in different age groups. About 11 to 26% of the population has dental fear and anxiety, which is due to the fear of injections [2]. Effective anesthesia is essential for a dental procedure. However, many patients' fear is due to injections, which, if not done properly, prevent pain control [3].

Pediatric behavioral control is essential in pediatric dentistry. Using vibration and cold at the infiltration site is a nonpharmacological method that reduces the infiltration pain [4].

According to gates control theory, the use of vibration during infiltration reduces pain, and the pain signal in the spinal cord is either blocked or transmitted to the spinothalamic fibers and then to the brain. When larger diameter fibers (A-beta fibers) such as mechanoreceptors are activated by pressure, ice packs, and vibration, the pain transmitted by A-delta fibers and C is blocked, and only the vibration sensation is interpreted. In addition, vibration causes the anesthetic fluid to enter the bloodstream faster and reduces the infiltration-induced swelling [5–7].

Cold is also an effective, convenient, and inexpensive factor for pain control. Cold slows or eliminates pain signal transmission. Studies have shown that cold increases the pain threshold against harmful stimuli such as needle penetration during local anesthetic infiltration [4, 8].

There are various devices for transmitting vibration to the injection site both internally and externally. The BUZZY device applies external cold and vibration to the desired area simultaneously. It has a bee-like plastic body that transmits vibration and a wing that is placed in the freezer, attached to the BUZZY body during use, and applies cold. The vibration component can be activated by a switch located on the top of the device. The ice wing component contains 18 grams of ice, which can be separated and stored in the freezer between stages. Each pair of wings can remain frozen at room temperature for about 10 minutes and can be used up to 100 times [9].

The BUZZY device has been used in the medical field to reduce pain and stress caused by intravenous injections and vaccines. Moadad et al. [10] investigated the effectiveness of using a BUZZY device when administered intravenously to children. The BUZZY device was significantly effective in reducing pain. Another randomized clinical trial in 2021 measured the effectiveness of the BUZZY device in reducing needle pain during vaccination in 30 patients in a hospital in France. The BUZZY device did not affect the needle-induced pain reduction [11]. A systematic review in 2020 on the best intervention to increase the acceptability of anesthetic injections in children concluded that more studies are needed, and it is still not possible to say with certainty which method is more acceptable [9].

To the best of the researchers' knowledge, there is not enough information available about the effectiveness of BUZZY in dentistry, and further studies are needed due to the conflicting results of the studies discussed. Therefore, the present study was conducted to investigate the effectiveness of cold and vibration in controlling pain during infiltration in children to improve the pain control methods and increase the children's satisfaction with treatment.

## 2. Method

### 2.1. Ethical Approval

The present double-blind randomized clinical trial was conducted in the pediatric ward of Isfahan Dental School from March to August 2021. This study was approved by the ethics code IR.MUI.RESEARCH.REC.1400.220 from Isfahan University of Medical Sciences and the IRCT code IRCT2021081505296N1 from the Ministry of Health of the Islamic Republic of Iran. Before the intervention, the procedure was fully explained to the parents, participation in the study was completely voluntary for the patients, and informed consent was taken from all the patients.

### 2.2. Participants

Thirty children aged 6–12 years were recruited for this study. They were physically and mentally healthy, did not take analgesics or sedatives, were positive or completely positive during the dental examination according to Frankel's behavioral scale, had no previous history of dental treatment, did not have toothache when they were referred, needed deep repair of maxillary *D* or *E* teeth, and were willing to participate in the study. Children with a history of hospitalization, chronic illnesses (such as asthma, allergies, diabetes, sickle anemia, cystic fibrosis, and dermatitis), behavioral problems (such as autism, hyperactivity disorder, and learning disabilities), mental problems, congenital disorders, and hearing and speech disorders were excluded from the study. The child was also excluded from the study if the area where the device was to be placed had pathology or if there was inflammation at the injection site. Noncooperative children were also excluded from the study.

### 2.3. Setting

In this study, the evaluator and participants were blinded to the experiment. In the first session, after examining and familiarizing the child with the dental environment and study equipment, consent was obtained from the parents, and age, sex, and Frankl's rating were included in the checklist. Then, using the envelope, it was randomly determined in which session the BUZZY device with the local anesthetic gel and in which session the local anesthetic gel and cold alone should be used. The type of intervention was written in two envelopes, and the child was asked to choose one of the envelopes. The envelope chosen by the child was considered the first session intervention. During the intervention session, the nurse placed the BUZZY device on the skin of the injection site on the same cheek and held it for two minutes. The pediatric dentist then applied 20% benzocaine local anesthetic gel with a cotton swab to the injection site mucosa, and after 30 seconds, performed infiltration anesthesia (1.8 ml lidocaine + 1.100,000 epinephrine) for one minute. The BUZZY device was in place during the application of local anesthesia gel and infiltration. The cold and local anesthetic gel was used in the control session. To make the child and the scorer of the pain behavioral scale blinded, the BUZZY device was placed on the child's face without turning on its vibration and the frozen wings. After applying the local anesthetic gel, infiltration anesthesia was performed for one minute.

### 2.4. Data Sources/Measurement

The pain was measured by the Wong–Baker subjective scale. Many children cannot explain and describe pain directly. The most reliable method to measure pain in children is self-report. Self-report is recognized as the gold standard for pain measurement in children. The Wong–Baker scale has been proven to be valid and reliable in the age group ≥4 years [12]. This scale has 6 painted faces with 10 points. The number 0 indicates no pain, and the number 10 indicates the most severe pain. Immediately after the infiltration, the child was asked to point to a face that was closer to his or her pain and discomfort. The face, legs, activity, cry, consolability (FLACC) objective scale was also used to measure pain.

For young children in difficult and stressful situations, it is better to measure their pain by observing their behavior. This scale has the necessary validity and reliability to measure pain in the age range of 5–16 years [13]. In this scale, the child's body postures, including face, legs, activity, cry, and consolability are scored from 0 to 2 according to the relevant table, and then, the scores are summed up. Finally, a score of 0–10 is assigned to this scale. Score 0 shows no pain, 1–3 shows mild pain, 4–6 indicates moderate pain, and 7–10 indicates severe pain.

To calculate and ensure blinding, a camera out of the child's sight was installed so that it always recorded video from an angle. A person outside the study field who was fully acquainted with the study watched the videos and scored them in two stages. In the first stage, the video sound was turned off, and the first three modes (face, legs, activity) were evaluated. In the second stage, the video sound was turned on, and the next two modes (cry and consolability) were evaluated. The physiological heart rate scale was also used to measure anxiety. The heart rate increases with a rise in anxiety [14]. A pulse oximeter was used to measure the heart rate. Heart rate was recorded 5 minutes before, during, and 5 minutes after infiltration.

### 2.5. Statistical Methods

The obtained data were fed into SPSS IBM 25 software. Because pain and anxiety do not follow a normal distribution, nonparametric tests such as Wilcoxon, Mann–Whitney, and *t*-test were used to evaluate the effectiveness of the BUZZY device.

## 3. Results

This study compared the effect of external cold and vibration and cold alone on the maxillary infiltration anesthetic pain in children aged 6–12 years. A total of 47 children were included in this study, 17 of whom were excluded due to nonreferral in the second session, so 30 children completed the two treatment sessions. In other words, 60 infiltration injections were performed. Of the participants, 15 received intervention in the first session and 15 in the second session. Children who received the first external cold and vibration and the second session of cold alone were included in group A, and those who received the first session of cold alone and the second session of external cold and vibration were included in group B.

The mean and standard deviation of Wong–Baker and FLACC scales in the intervention and control sessions in groups A and B are presented in Table 1.

### 3.1. Wong–Baker Score Results

The mean total Wong–Baker score was 2.53 ± 2.72 in the intervention group and 2.73 ± 3.03 in the control group. The Mann–Whitney test was also used to evaluate the effectiveness of the BUZZY device. In this test, the Wong–Baker score difference between the intervention and control sessions showed no significant difference between the intervention and control groups (*P* value = 0.582). In addition, precedence and delay in using the BUZZY device did not cause a significant change in pain reduction (*P* value = 0.324).

### 3.2. FLACC Score Results

The mean total FLACC score was 1.90 ± 1.97 in the intervention group and 1.56 ± 1.77 in the control group. Further, the results of the Mann–Whitney test showed a significant difference in the FLACC score between the intervention and control groups (*P* value = 0.040). Therefore, according to the FLACC scale, the use of external cold and vibration has significantly reduced pain in children. Mann–Whitney test was also performed to evaluate the effect of precedence and delay in using the BUZZY device using the FLACC scale, which indicated no significant difference between the intervention and control groups (*P* value = 0.947).

### 3.3. Heart Rate Results

The mean heart rate differences before (PR0), during (PR1), and after (PR2) infiltration in the intervention and control sessions by gender are presented in Table 2. The Student's *t*-test indicated no difference in heart rate between groups A and B, and precedence and delay in the intervention had no significant effect on the heart rate reduction.

## 4. Discussion

Dental fear and anxiety are major reasons for failure to care for teeth and can negatively affect the overall oral health of the patient. The main reason for dental fear and anxiety is the fear of infiltration. Therefore, appropriate methods should be used to reduce infiltration pain to prevent patients from avoiding dental treatment. Pharmacological and nonpharmacological methods have been suggested to control infiltration pain in children. Pharmacological methods include sedation, anesthesia, general anesthesia, and the use of benzodiazepines and nitrous oxide. Nonpharmacological methods include playing, tell-show-do technique, parental presence, distraction, hand-over-mouth technique, music,. [15]. Studies have shown that the use of nonpharmacological behavioral control techniques is the gold standard for managing children experiencing needle pain [16]. The BUZZY device can be used to achieve this goal. It is fast, noninvasive, inexpensive, and reusable, and applies external cold and vibration to the target site [17].

The free nerve ending is present in all layers of the mucosa, including the epithelium. Cold exerts its effect by slowing down the signal conduction speed. Although different types of nerve fibers are guided at different rates, at each degree of temperature decrease, the signal conduction velocity is relatively reduced and stopped completely in the range of 0–10°C. Cooling the muscle tissue also reduces the activity of the muscle spindles and reduces their tone. The topical application of cold stimulates A-myelinated fibers and activates pain control pathways, which in turn increases the pain threshold [4, 8].

The results of the present study showed that the use of the BUZZY device effectively reduced pain during maxillary infiltration in children aged 6–12 years compared to the use of cold alone. However, the BUZZY device was not effective in reducing anxiety and stress. Studies by Alanazi et al., [14] Sahithi et al., [17], and Hegde et al. [18] have shown that cold and vibration can reduce pain and stress during infiltration.

Dental fear is a multidimensional factor that includes social, mental, and physiological dimensions and the use of one parameter is not sufficient to measure pain and fear. To accurately measure pain and anxiety during infiltration, it is better to use the three parameters of pain measurement, namely, self-report, behavioral, and physiological parameters. Self-reported assessment of pain, such as the Wong–Baker scale, enables the child to provide an immediate emotional response to dental treatment, which is an appropriate subjective criterion for reporting pain by the child [17].

In this study, the mean pain score was lower in the cold and vibration session than in the control session in groups A and B, but no statistically significant difference was observed between the groups. This is one of the drawbacks of a scale with faces that show the amount of pain more than it is. In a clinical trial with a parallel control, Suohu et al. [16] concluded that the Wong–Baker scale did not show a significant difference between the control and intervention groups, which was attributed to the child's tendency to choose higher-scale faces due to disappointment with dental procedures. Due to individual differences between patients, future studies are recommended to ask the child to score this scale before infiltration. These results contradict those of Sahithi et al.'s [17] study, in which 100 children aged 4–11 years who needed endodontic treatment or extraction were recruited. Wong–Baker and VAS scales were used to measure pain, and heart rate was used to measure anxiety. In this study, contrary to the present study, the Wong–Baker scale was significantly reduced in the BUZZY group. Moreover, in the study of Alanazi et al. [14], on 60 7-year-old children, the Wong–Baker scale was significantly reduced in the group receiving cold and vibration. Alanazi et al. and Hegde et al. [14, 18] also found similar results by examining the effects of cold and vibration on 30 children. Needle-related actions in children can become a conditioned stimulus. This can cause a great reaction in the child even with a gentle touch of the needle, and the amount of discomfort the child shows with his behavior may not be directly associated with the amount of injury.

The FLACC scale is a behavioral evaluation criterion that is used to assess pain in children and adults with cognitive impairment and severe illnesses [13]. The FLACC scale can be used to score a child's behavior during the procedure. In the present study, the mean FLACC score was significantly lower in the intervention session than in the control session. These results are consistent with the findings of Suohu, Alanazi, and Manasa Hegde, which indicated the control group had a higher FLACC score than the intervention group [14, 16, 18]. In Suohu et al.'s [16] study on 50 children aged 5–10 years, as in the present study, only the FLACC scale showed a significant difference between the intervention and control groups, and the researchers concluded that the use of external vibration was better than conventional anesthesia in reducing pain. In the study of Alanazi et al. [14] on 60 children, one group received external cold and vibration, and the other group received conventional anesthesia. In this study, the FLACC and Wong–Baker scales and heart rate indicated lower scores in the intervention group than in the control group. The authors concluded that vibration and cold reduce the children's discomfort and fear during infiltration.

A high heart rate indicates the presence of pain. Increased heart rate is also directly related to stressful conditions [14]. That is why heart rate was used to measure stress in the present research. The mean heart rate difference before and during injection was not significantly reduced in either group A or group B in either session. These results are in line with the findings of Kalpna Chaudhry's study in which 20 children aged 8–14 years were studied. In the first session, conventional anesthesia was performed, and in the second session, anesthesia was performed along with VibraJect. In this study, stress measurement criteria, including heart rate, blood pressure, and temperature were not significant unlike pain measurement criteria [19]. Moreover, in Suohu et al.'s [16] study on 20 children aged 5–10 years, heart rate changes were not significant contrary to the FLACC criteria. However, the results of the present study contradict those of Alanazi et al. and Sahithi et al. [14, 17]. Alanazi et al.'s [14] study with a split-mouth design was performed on 60 children. The heart rate was higher in the control group than in the intervention group. The difference in results is probably because they measured heart rate only during the infiltration, while each person's basal heart rate differs from the other. This difference may also be due to the larger sample size in their study.

One of the limitations of this study is the low number of participants due to the lack of patients for the second treatment. The results of this study are only reported for cooperative children and infiltration injections and cannot be generalized to noncooperative children who need other injections and are less than 6 years old. The person who recorded the child's heart rate and pain level was not blinded to the study. Future studies are suggested to recruit more participants. It is also a good idea to ask children to score the Wong–Baker scale before infiltration. More scales such as SEM, which is a behavioral scale, and Visual Analog Scale (VAS) can also be used. More comprehensive results will be obtained if the parents and the doctor are also asked to rate the child's behavior or pain during infiltration. It is also recommended to compare the BUZZY device with other devices such as VibraJect and DentalVibe.

## 5. Conclusion

Within the limitations of this study, the use of the BUZZY device can be effective in reducing the maxillary infiltration-induced pain in children aged 6–12 years compared to the use of cold alone. However, the BUZZY device was not effective in reducing anxiety and stress. Moreover, using the device in the first or second session did not affect pain reduction.

## Figures and Tables

**Table 1:** Mean and standard deviation of Wong-Baker and FLACC scales in groups A and B.

FLACC				Wong–Baker			
B		A		B		A	
Cold	Cold and vibration	Cold	Cold and vibration	Cold	Cold and vibration	Cold	Cold and vibration
1.22 ± 1.06	2.03 ± 2.00	2.12 ± 2.06	1.97 ± 1.80	2.75 ± 2.80	2.15 ± 2.93	3.39 ± 2.66	3.22 ± 2.13

**Table 2:** Mean difference of heart rate (before and during) and (before and after) infiltration in groups A and B.

	Method used in the first session			
	A		B	
	Cold and vibration	Control	Cold and vibration	Control
PR1-PR0	3.87	2.47	3.00	−2.40
PR2-PR0	1.87	1.74	1.13	−0.40

## Data Availability

The datasets used and/or analyzed during the current study are available from the corresponding author on reasonable request.
